# The Assessment of Carbon Dioxide Dissociation Using a Single-Mode Microwave Plasma Generator

**DOI:** 10.3390/molecules25071558

**Published:** 2020-03-28

**Authors:** George Mogildea, Marian Mogildea, Cristina Popa, Gabriel Chiritoi

**Affiliations:** 1 Institute of Space Science, 077125 Magurele, Romania; george_mogildea@spacescience.ro (G.M.); gabriel.chiritoi@spacescience.ro (G.C.); 2 National Institute for Laser, Plasma and Radiation Physic, Laser Department, 409 Atomistilor St., PO Box MG-36, 077125 Magurele, Romania; cristina.achim@inflpr.ro

**Keywords:** assessment of gases, carbon dioxide dissociation, plasma generator, OES spectroscopy method

## Abstract

This paper focuses on the dissociation of carbon dioxide (CO_2_) following the absorption processes of microwave radiation by noncontact metal wire (tungsten). Using a microwave plasma generator (MPG) with a single-mode cavity, we conducted an interaction of microwaves with a noncontact electrode in a CO_2_ atmosphere. High energy levels of electromagnetic radiation are generated in the focal point of the MPG’s cylindrical cavity. The metal wires are vaporized and ionized from this area, subsequently affecting the dissociation of CO_2._ The CO_2_ dissociation is highlighted through plasma characterization and carbon monoxide (CO) quantity determination. For plasma characterization, we used an optical emission spectroscopy method (OES), and for CO quantity determination, we used a gas analyzer instrument. Using an MPG in the CO_2_ atmosphere, we obtained a high electron temperature of the plasma and a strong dissociation of CO_2_. After 20 s of the interaction between microwaves and noncontact electrodes, the quantity of CO increased from 3 ppm to 1377 ppm (0.13% CO). This method can be used in space applications to dissociate CO_2_ and refresh the atmosphere of closed spaces.

## 1. Introduction

The exploration of the Universe has been an important goal of international space programs for decades. The building of the International Space Station (ISS) is the most important engineering feat of humankind, as its existence provides the opportunity to consider further exploration of other planets. NASA and its international partners have recently begun to plan the exploration of Mars, with the hope of bringing new opportunities and knowledge to Earth. Currently, all life-sustaining resources for the ISS are from Earth. Therefore, in order to carry out long-term interplanetary missions, it is necessary to develop new oxygen recycling technologies for astronauts’ breathing apparatus. In such conditions, the dissociation of CO_2_ using plasma systems may be a good choice.

Plasma is a good environment for CO_2_ dissociation because it contains high energy electrical charges. The disintegration process of gas molecules from plasma is based on the collisions between charged species created in the plasma and neutral particles.

In order to refresh the air inside the spacecraft, electrical energy sourced from its solar panels or nuclear systems can be used to power the plasma systems.

Amongst the different experimental plasma systems developed, the best-known systems are: dielectric barrier discharges (DBD) [1,2], inductively coupled radiofrequency plasma [3,4], microwave discharges [3,5,6], glow discharges [7,8] and gliding arc discharge reactors [9].

Experimental studies show that the conversion rate efficiency of CO_2_ depends on the temperature of the plasma. The energy required to start the CO_2_ dissociation process is in accordance with Formula (1) [9]. The endothermic plasma-chemical process of CO_2_ is

(1) $${\mathrm{CO}}_{2}\to \;\mathrm{CO}+1/2O_{2},\;\Delta H=2.9\;\mathrm{eV}/\mathrm{molecule}$$

The energy required to start the process of total decomposition of CO_2_ is displayed in Formula (2) [9].

(2) $${\mathrm{CO}}_{2}\to \;\mathrm{CO}+O,\;\Delta H=5.5\;\mathrm{eV}/\mathrm{molecule}$$

The CO_2_ molecule begins to split into CO and O_2_ near 2000 K. The decomposition of CO_2_ can only be carried out at high temperatures (3000–3500 K) [10]. The conversion rate efficiency of CO_2_ at a plasma temperature of 2000K is very small (<1%), and at a plasma temperature of 3500K it is 60% [11]. Conversion rates vary between plasma system types, from less than 4% for DBD [12], to less than 35% for microwave discharges and gliding arc discharges [6,12].

The gliding arc discharge reactor is attracting considerable attention because it can create both nonthermal and thermal plasmas [11]. The electron temperature of the plasma can rise up to 10,000–23,000 K in a gliding arc discharge reactor (approximately 2 eV). [11]

The microwave discharge method is interesting from a scientific point of view, because the microwaves are non-ionizing-radiation and the microwave photons do not have enough energy for the ionization of CO_2_ molecules. Microwave CO_2_ dissociation is deemed high in efficiency due to vibrational excitation, as this results in a strong nonequilibrium between vibrational and translational degrees of freedom, which in turn intensifies dissociation. [13] Microwave discharge systems use microwaves to create plasma from the CO_2_ atmosphere and to dissociate CO_2_, while the gliding arc discharge method uses an electric current and electrodes to ionize and dissociate CO_2_.

New methods with more efficient conversion rates are required. Therefore, our research is focused on the dissociation of CO_2_ produced by the interaction between a microwave field and a noncontact metallic electrode of an MPG. Our method is based on the combination of the gliding arc discharge and the microwave discharge methods. To our knowledge, this is the first time that the CO_2,_ dissociation process using plasma resulting from the interaction between metal wires and microwaves is carried out.

The physical processes involved in microwave interaction with matter are very different. Dielectric materials can volumetrically absorb the electromagnetic energy from the microwave and transform it into heat [14]. Research concerning microwave applications for the heating of metal is seldom completed, as it is well-known that bulk metals reflect microwave radiation, and thus cannot be directly heated [15]. Microwave interaction with metal is restricted to its surface only. Studies from 2001 revealed that metallic powders (tungsten carbide–cobalt) introduced into a multimode cavity in a microwave field can absorb microwave radiation [16].

Other studies conducted in 2009 [17] highlighted that microwave absorption depends on the dimensions of the metallic particle and the frequency of the electromagnetic radiation. In 2009 A. Mondal et al. exposed Cu powder (particles between 6–385 μm) to microwaves in a multimode cavity for 10 min. The experimental results showed that the Cu powder particles with dimensions of 6 μm reached temperatures of about 1000 °C, and that microwave absorption of the metallic powders depended on the depth of penetration.

Other recent studies showed that the microwave absorption process can be present in the interaction of nonionizing radiation with metallic objects that have dimensions much larger than the depth of penetration [18].

Using an MPG with a single-mode cylindrical cavity in 2010 [18], we demonstrated that lead metal wires with diameters less than 0.5 mm can be vaporized and ionized by microwaves. The experiment unfolded in the air at atmospheric pressure. Later, we investigated the possibility of controlling the quantity of metal (lead wires) which are vaporized and ionized in this manner [19].

In 2010 we also investigated [20] the microwave heating behaviours of metal wires (lead) in vacuum conditions. The same MPG was used. We found that at 10^−5^ millibar pressure, the vaporization and ionization processes of the metal wires did not occur, and the metal wires were only heated up to melting temperature. Thus, metal wires can be heated up to plasma temperature in air conditions at a normal atmosphere and at room temperature, but in vacuum conditions metal wires do not exceed the melting temperature. However, it is possible that air molecules could contribute to the microwave absorption process of metal. In order to investigate whether gas molecules contribute to the microwave heating process, we used metal wire together with a small Teflon piece. The main conclusion of the experiment was that if a metal wire and Teflon piece are exposed to a microwave field, the metal wire generates a strong electric field and the Teflon is heated and decomposed into a gas [21]. This electric field ionizes the Teflon gas, and the heat subsequently generated by the Teflon ion gas is able to vaporize the metal wire. The metal vapors are then ionized in the cylindrical cavity’s microwave electric field.

This process is explained by to the Poynting equation displayed in Equation (3) [22].

(3) P→=12Re(E→×H∗→)=12|E|2η

where, *P* = Poyting vector, *E* = electric field, *H* = magnetic field, *η* = impedance of medium.

If the microwave power in the cylindrical cavity increases, the electric field in the metal wire also increases. The microwaves induce strong electric fields in metal wires (3 MV/m to 6 MV/m [23]). The high electric field causes the ionization of the gas surrounding the metal wire in the high-intensity area. The ions of the plasma interact with the metal wire and vaporize it.

The relationship between the microwave electric field and the current density (conduction and displacement current) of the metal wire is [24,25]:

(4) $$J=\left(\sigma +j\omega \varepsilon \right)\vec{E}$$

where: J = current density in metal wires, *σ* = electrical conductivity of metal wires, *ω* = angular frequency, *ε* = electrical permittivity, *E* = electric field.

Other similar experiments were conducted in 2015, in which Popescu S. et al. created titanium plasma from titanium samples [26] in the air at atmospheric pressure. A microwave device with a rectangular cavity was used in the experiment. The plasma column was created when a titanium electrode irradiated by microwaves was brought into contact with a titanium source plate. These studies showed that metallic vapor and OH radicals were generated in an air atmosphere.

The current objective of this research is the investigation of the dissociation of carbon dioxide (CO_2_) following the absorption processes of microwave radiation by a tungsten (W) wire.

In the experiment, we used an MPG in order to generate the plasma from a noncontact tungsten electrode. The experiment was conducted in a CO_2_ atmosphere.

The optical emission spectroscopy method (OES) was used to identify the atomic species and determine the plasma’s electron temperature. The CO quantity in the CO_2_ atmosphere was analyzed using a gas analyzer kit instrument.

## 2. Materials and Methods

**Figure 1:**
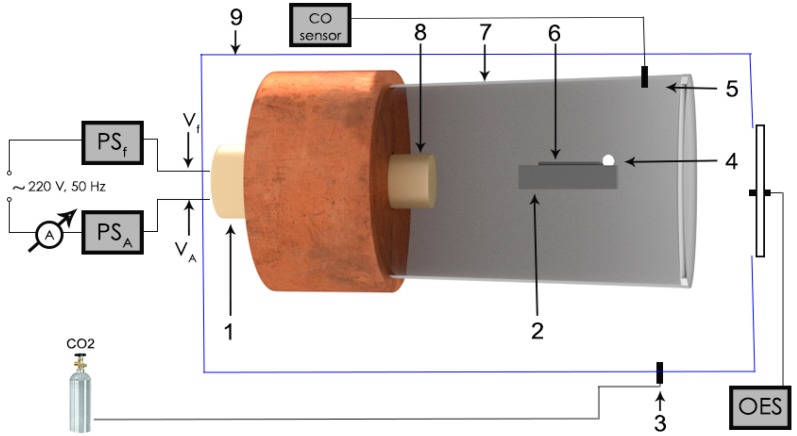
Experimental design of the CO_2_ dissociation: 1—magnetron, 2—ceramic support, 3—CO_2_ connector, 4—high electromagnetic energy region, 5—CO_2_ sensor, 6—metallic wire, 7—cylindrical cavity, 8—magnetron antenna, 9—pressure chamber.

The dissociation of CO_2_ molecules is based on the interaction between microwave fields and a metal wire in a CO_2_ atmosphere. We used an MPG to generate plasma from a noncontact electrode. The MPG was developed by our team. The microwave plasma generator was introduced into the pressure chamber with a CO_2_ atmosphere. The volume of the pressure chamber was 0.024 m^3^ (24 L) as shown in Figure 1.

The microwave plasma generator consists of a cylindrical cavity, a microwave source (commercial magnetron) with ν = 2.45 GHz, 800 W emitted power and a power supply (one high voltage power supply for the magnetron anode—PSA, and one low voltage power supply for the filament of the thermoelectronic source of the magnetron—PSF). The PSA contains an electronic module to modify the length of the electric impulses (duty factor) of the source that feeds the anode magnetron. Therefore, by changing the duty factor of the anode voltage from the magnetron, we modified the quantity of the vaporized metal and obtained different quantities of dissociated CO_2_ as a result.

Cylindrical cavity: the TM cylindrical cavity dimensions were calculated with Formula (5) [22,24].

(5) $${\left(f_{r}\right)}_{mnl}^{TM}=\left(\frac{1}{2\pi \sqrt{\mu \cdot \varepsilon }}\right)\cdot \sqrt{{\left(\frac{p_{01}}{a}\right)}^{2}+{\left(\frac{l\pi }{h}\right)}^{2}}$$

*a*—radius of the cylindrical cavity (m);
*h*—height of the cylindrical cavity (m);
*l*—Longitudinal mode of the cavity;
*μ*—Permeability of the medium within cavity (H/m);
*ε*—Permittivity of the medium within the cavity (F/m);
*p*_01_—First zero of the Bessel function (equal to approximately 2.405);
*f_r_*—The resonant frequency of the cavity.

The indices mnl of the TM propagation mode refer to the number of half-wavelength variations in the radial, axial and longitudinal directions. The optimal dimensions of the *TM*_011_ cylindrical cavity are a diameter of 11 cm and a length of 10.5 cm.

**Figure 2:**
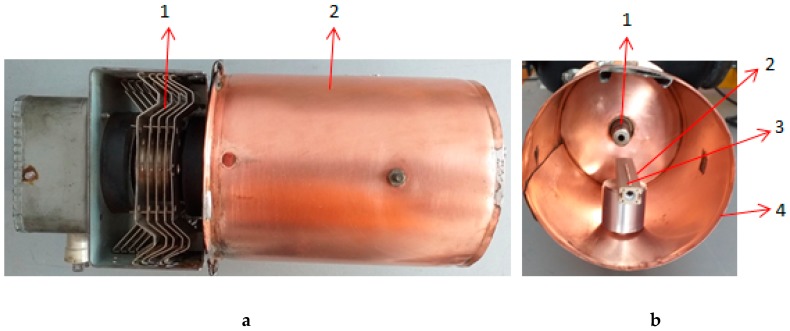
The microwave plasma generator: (**a**) 1—magnetron, 2—cylindrical cavity lateral view; (**b**) 1—magnetron antenna, 2—ceramic support, 3—metallic wire; 4—cylindrical cavity front view; (**a**) Lateral view (**b**) Front view.

The first preparation step of the experiment was to introduce the noncontact electrode into the cylindrical cavity shown in Figure 2. As the microwave electric field propagates along the axis of the cavity in the *TM*_011_ propagating mode, the metallic electrode must be positioned along, and as close as possible to the axis.

The high-density energy region (focal point) is located 6.1 cm from the magnetron antenna, which corresponds to half-wavelength for the 2.45 GHz microwave frequency. One end of the metallic electrode was placed in the focal point of the cylindrical cavity. The noncontact electrode used was a tungsten wire with a diameter of 0.5 mm and a length of 50 mm. The sample was placed on a ceramic support and introduced into the cavity shown in Figure 2.

**Figure 3:**
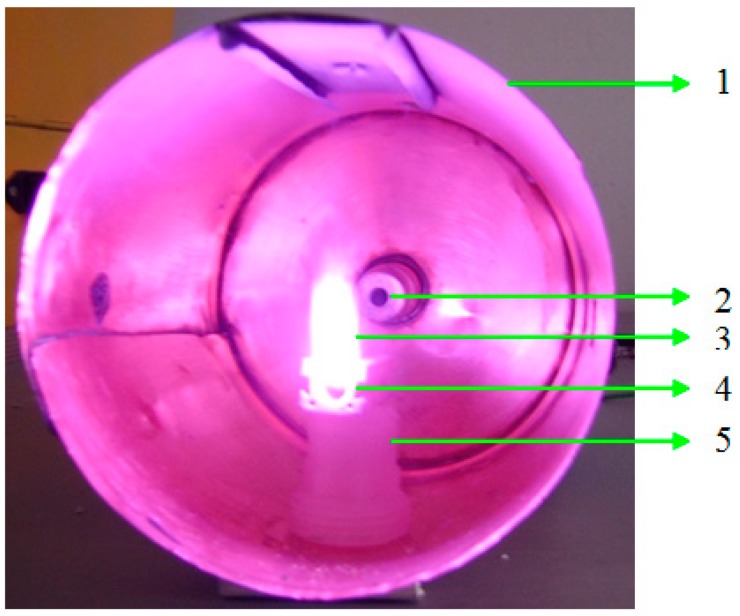
The metallic wire exposed to the microwave field: 1—cylindrical cavity, 2—magnetron antenna, 3—metallic plasma, 4—ceramic support, 5—plastic support.

The pressure chamber was filled with CO_2_ at atmospheric pressure, and the microwave source was turned on. In these conditions, the tungsten wire was exposed to microwaves for 20 s. As displayed in Figure 3, the plasma was created at 700 W microwave power in a CO_2_ atmosphere, at normal atmospheric pressure and ambient temperature.

The tungsten wire was partially vaporized from this area, as explained in Table 1. We chose tungsten (W) as a noncontact electrode because this metal has high electrical conductivity which leads to a small rate of metal vaporization [26] as shown in Table 1. The tungsten wire was slowly consummated during the CO_2_ dissociation process, which allowed us to dissociate CO_2_ for a long time.

Following the interaction between the microwave and the tungsten electrode, we analyzed the plasma components and we determined the CO quantity in the CO_2_ atmosphere.

For plasma characterization, we used the optical emission spectroscopy method (OES) [27,28].

Using the Ocean Optics USB 2000 spectrometer, we recorded the optical emission spectrum of the plasma with an integration time of 1ms.

To determine the CO concentration in the CO_2_ atmosphere, we used the CHEMIST 504S gas analyzer kit instrument, which is an instrument that uses an electrochemical sensor to measure the concentration of CO with a resolution of 2 × 10^4^ ppm.

## 3. Results and Discussion

Microwave plasma is widely investigated because of its potential to provide highly efficient CO_2_ conversion. Thus far, various experimental microwave devices have been developed. All microwave devices create plasma when microwaves interact directly with a CO_2_ atmosphere. The dissociation of CO_2_ is based on the microwave absorption process of CO_2_. The value of the electron temperature of the plasma obtained with the actual microwave devices does not exceed 7000 K [5,10].

In our experiment, the dissociation of CO_2_ was based on the microwave absorption process by metals. This physical process is characterized by Ohm losses [17]. The Ohm loss process is present when a metal wire is crossed by an electric current, or in our experiment, when a noncontact electrode interacts with microwaves.

Therefore, when microwaves interact with a noncontact electrode, a high electric field (3 MV/m–6 MV/m) is generated in the focal point of the cavity, as shown in Figure 1. When the noncontact electrode comes into contact with the gas atmosphere, the electrode is vaporized and ionized, and the gas is also ionized. The dissociation of CO_2_ molecules is produced in two ways: the collision of CO_2_ molecules with electrical charges generated by microwave in a noncontact electrode, and the interaction between the metal’s ions and CO_2_ molecules. In 20 s of interaction between the noncontact electrode, CO_2_, and the microwave field, the tungsten electrode was partially vaporized and ionized.

**Figure 4:**
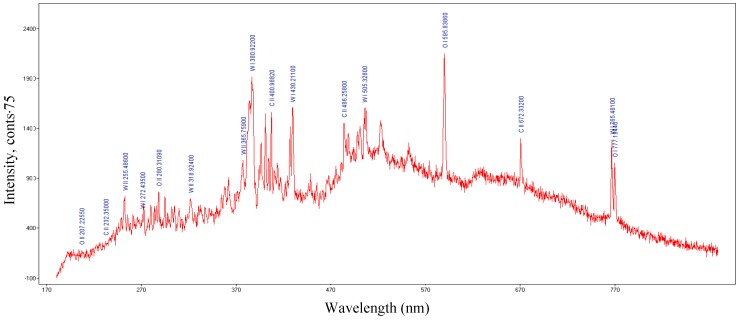
Optical emission spectra of the plasma: Ions generated during CO_2_ dissociation process.

**Figure 5:**
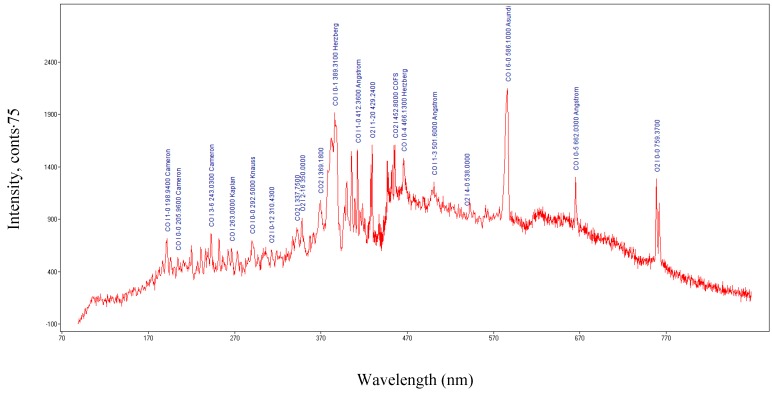
Optical emission spectra of the plasma: The monoxide and oxygen generated during the CO_2_ dissociation process.

Metallic vapors and ions of the tungsten and ions of the gases such as CO, O_2_, O, C, etc., were generated in the cylindrical cavity, as displayed in Figure 4 and Figure 5.

The ions were highlighted using the optical emission spectroscopy method (OES) [27,28].

Spectrum Analyzer software [29] was used to identify optical emission spectra of the plasma. The microwave field created ions of metal, oxygen, and carbon. The ions were observed from the optical emission spectra, shown in Figure 4. Ions of oxygen (OI and OII), carbon (CII) and tungsten (WI and WII) were observed in the plasma emission spectra, displayed in Figure 4. During the CO_2_ dissociation process, other gas molecules such as CO and molecular oxygen were formed, as shown in Figure 5 [30,31,32].

**Figure 6:**
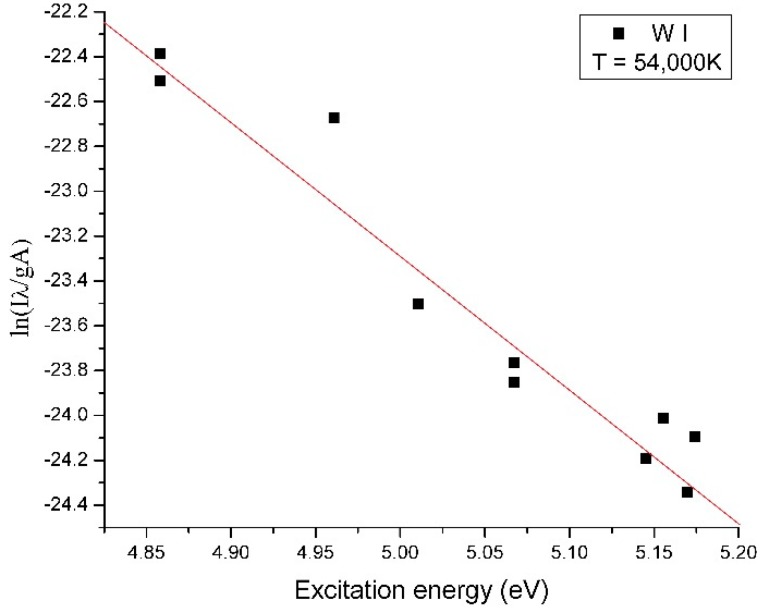
The Boltzmann plot and electron temperature for tungsten ions.

**Figure 7:**
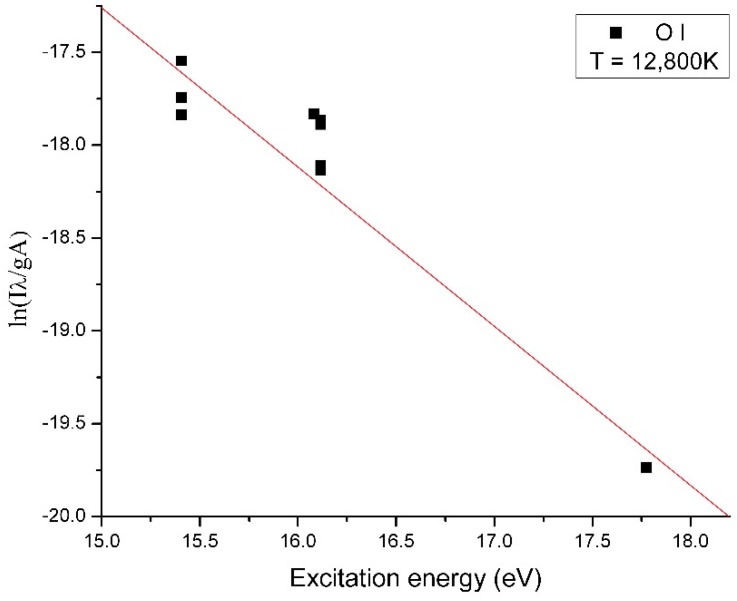
The Boltzmann plot and electron temperature for ions oxygen.

The monoxide and molecular oxygen are highlighted in the optical emission spectra of the plasma in Figure 5. To assess the CO_2_ dissociation efficiency of our method, we determined the electron temperature of the plasma. The electron temperature of tungsten plasma and oxygen plasma was determined using the Boltzmann plot method, which assumes that local thermodynamic equilibrium (LTE) is met within the plasma. The electron temperature for tungsten ions was 54,000 K, and the electron temperature for oxygen ions was 12,800 K as shown in Figure 6 and Figure 7, respectively.

We measured the CO in the CO_2_ atmosphere before both starting and stopping the microwave discharge.

The following parameters were recorded: before creating plasma, the concentration of the CO in the CO_2_ atmosphere was 3 ppm.

After the 20 s reaction time, (interaction time between the microwave field and metal wire), the quantity of the CO generated during the CO_2_ dissociation process was increased to 1377 ppm.

## 4. Conclusions

Using a microwave plasma generator, we highlighted that CO_2_ was dissociated through interaction between metal wires and the microwave field.

During the interaction between the microwave and tungsten wire, thermal plasma and non-thermal plasma were generated.

We obtained a high value for the electron temperature of the plasma (54,000 K or 4.7 eV for WI and 12,800 K or 1.1 eV for OI).

During interaction with the plasma, ions of OII, WII, CII and gases such as CO and O_2_ were created.

After 20 s reaction time, the quantity of CO increased from 3 ppm to 1377 ppm inside the pressure chamber, meaning that 0.13% CO was generated in 24 L of CO_2_ in this period.

Using our method, we obtained a high electron temperature of the plasma, and subsequently a better CO_2_ dissociation efficiency.

## Figures and Tables

**Table 1:** Energetic parameters and vaporization rate of the tungsten.

Tungsten Wire	First Ionization Energy (eV)	Vaporization Rate (mg/s)	Electrical Conductivity (S/m)
(0.5 mm diameter)	7.98	2	2 × 10^7^
